# In-situ gelation of fibrin gel encapsulating platelet-rich plasma-derived exosomes promotes rotator cuff healing

**DOI:** 10.1038/s42003-024-05882-7

**Published:** 2024-02-20

**Authors:** Molin Li, Lin Shi, Xianghui Chen, Dan Yi, Yufei Ding, Jian Chen, Guanghui Xing, Siming Chen, Li Wang, Yongyi Zhang, Yaqiong Zhu, Yuexiang Wang

**Affiliations:** 1 Medical School of Chinese PLA, Beijing, China; 2 Department of Ultrasound, The First Medical Center, Chinese PLA General Hospital, Beijing, China; 3 State Key Laboratory of Kidney Diseases, National Clinical Research Center for Kidney Diseases, Beijing Key Laboratory of Kidney Disease Research, Beijing, China; 4 Beijing Friendship Hospital, Capital Medical University, Beijing, China; 5 School of Medicine, Nankai University, Tianjin, China; 6 Department of Ultrasound, the Fourth Medical Center, Chinese PLA General Hospital, Beijing, China; 7 No. 962 Hospital of the PLA Joint Logistic Support Force, Harbin, China

**Keywords:** Cell growth, Molecular biology, Regeneration

## Abstract

Although platelet-rich plasma-derived exosomes (PRP-Exos) hold significant repair potential, their efficacy in treating rotator cuff tear (RCT) remains unknown. In light of the potential for clinical translation of fibrin gel and PRP-Exos, we evaluated their combined impact on RCT healing and explored suitable gel implantation techniques. In vitro experiments demonstrated that PRP-Exos effectively enhanced key phenotypes changes in tendon stem/progenitor cells. Multi-modality imaging, including conventional ultrasound, shear wave elastography ultrasound, and micro-computed tomography, and histopathological assessments were performed to collectively evaluate the regenerative effects on RCT. The regenerated tendons exhibited a well-ordered structure, while bone and cartilage regeneration were significantly improved. PRP-Exos participated in the healing process of RCT. In-situ gelation of fibrin gel-encapsulated PRP-Exos at the bone-tendon interface during surgery proved to be a feasible gel implantation method that benefits the healing outcome. Comprehensive multi-modality postoperative evaluations were necessary, providing a reliable foundation for post-injury repair.

## Abstract (web summary)

In this study, the authors have investigated if the implantation of fibrin gel loaded with platelet-rich plasma-derived exosomes is a feasible strategy to treat rotator cuff injuries. In fact, this strategy proved to promote tendon repair and healing outcomes in a rabbit model of rotator cuff injury.

## Introduction

Rotator cuff tear (RCT) is a prevalent orthopedic condition, often resulting in pain, weakness, and restricted functionality among patients^1^. In recent years, arthroscopic rotator cuff repair has emerged as a widespread and effecacious surgical treatment in clinical practice^2^. However, previous studies have indicated that the remodeled bone-tendon interface (BTI) following surgery tends to develop disorganized scar tissue due to inadequate blood supply and a lack of functional cells. This scar tissue exhibits low compliance and weak mechanical stress, which is closely associated with postoperative re-tear rates ranging from 27 to 94%, along with the occurrence of other clinical complications^3^. Currently, a significant amount of research focuses on treatment methods based on stem cell therapy, growth factor therapy, and functional tissue engineering techniques involving biomaterials to address the regenerative requirements of the BTI. However, concerns regarding safety pose significant challenges to the clinical translation of these approaches^4–6^.

Exosomes are bilayer nanoscale vesicles (~20–150 nm in diameter) that are secreted by cells. They exhibit stability and low immunogenicity and serve as carriers of various biological information, facilitating intercellular communication and enabling cross-species information transmission^7^. At present, emerging cell-free therapies based on mesenchymal stem cell-derived exosomes have made significant strides in tissue engineering development. However, the in vitro expansion of stem cells poses challenges, resulting in low exosome yields and high costs, impeding clinical translation. Platelet-rich plasma (PRP) represents a high-concentration platelet product known for its safety, standardized preparation, and relatively affordable cost^8^. PRP has demonstrated potent abilities to promote tissue regeneration and repair in a few domains, including the musculoskeletal system, nerve regeneration, and wound healing^9–11^. However, PRP exhibits several limitations in clinical applications. Its repeatability is compromised due to individual variations among donors and the influence of storage conditions. Furthermore, the absence of unified quality control standards for PRP results in substantial heterogeneity in the obtained concentrates^12^.

Compared to the utilization of PRP or exosomes derived from stem cells, PRP-derived exosomes (PRP-Exos) offer several distinct advantages. PRP-Exos are safe, and platelets lack nuclei, which serves to mitigate potential oncogenic risks^13^. Moreover, utilizing PRP as the source of exosomes mitigates the risk of contamination that may arise during the in vitro expansion phase of mesenchymal stem cells. This approach enhances quality control in production and addresses regulatory issues^14^. In addition, the preparation of PRP-Exos is cost-effective, circumventing the need for specialized equipment and manpower required in the GMP cell culture process. Consequently, this reduces the operational costs associated with clinical applications^15^. Finally, surplus clinical-grade allogeneic platelets represent a readily available national resource. PRP-Exos, which can be abundantly generated from autologous patient platelets or other discarded allogeneic platelet units, demonstrate minimal batch-to-batch variability in growth factor levels^16,17^. This characteristic offers greater opportunities for regulatory oversight and widespread clinical application, potentially overcoming the limitations inherent in the clinical translation of PRP and exosomes sourced from other sources.

In addition, PRP-Exos are rich in bioactive factors^16,18^. Their clinical application value is not only reflected in the coagulation function similar to PRP but PRP-Exos can also traverse various tissue barriers. Notably, they possess the ability to promote re-epithelialization and angiogenesis^18–21^ and expedite the regeneration of bone and cartilage^22,23^. These findings underscore the substantial promise of PRP-Exos in the treatment of RCT.

Owing to the rapid clearance limitation associated with the injection of PRP-Exos into the rotator cuff, we opted for the use of readily available fibrin gel (FG) as a biological scaffold for encapsulating exosomes, thereby stabilizing their bioactivity. FG is approved by China’s NMPA (approval number S20030070), exhibits excellent biocompatibility and a predictable degradation period, and can assist in hemostasis and tissue sealing. The method of gel implantation is a critical step in ensuring effective repair. While several studies have employed a post-in vitro solidification approach for gel implantation gel implantation^24^, only a few studies have attempted in situ gelation at the BTI during surgery.

In this study, we employed FG to encapsulate PRP-Exos, resulting in the development of a gel sustained-release system (FG-PRP-Exos). Our study has revealed that the in situ gelation of FG-PRP-Exos during surgery is feasible, and the use of multi-modality evaluation techniques provides a reliable basis for assessing therapeutic efficacy in the repair of RCT. These efforts serve as a foundational basis for advancing the clinical translation of PRP-Exos.

## Results

### Identification and characterization of PRP-Exos and tendon stem/progenitor cells

**Fig. 1:**
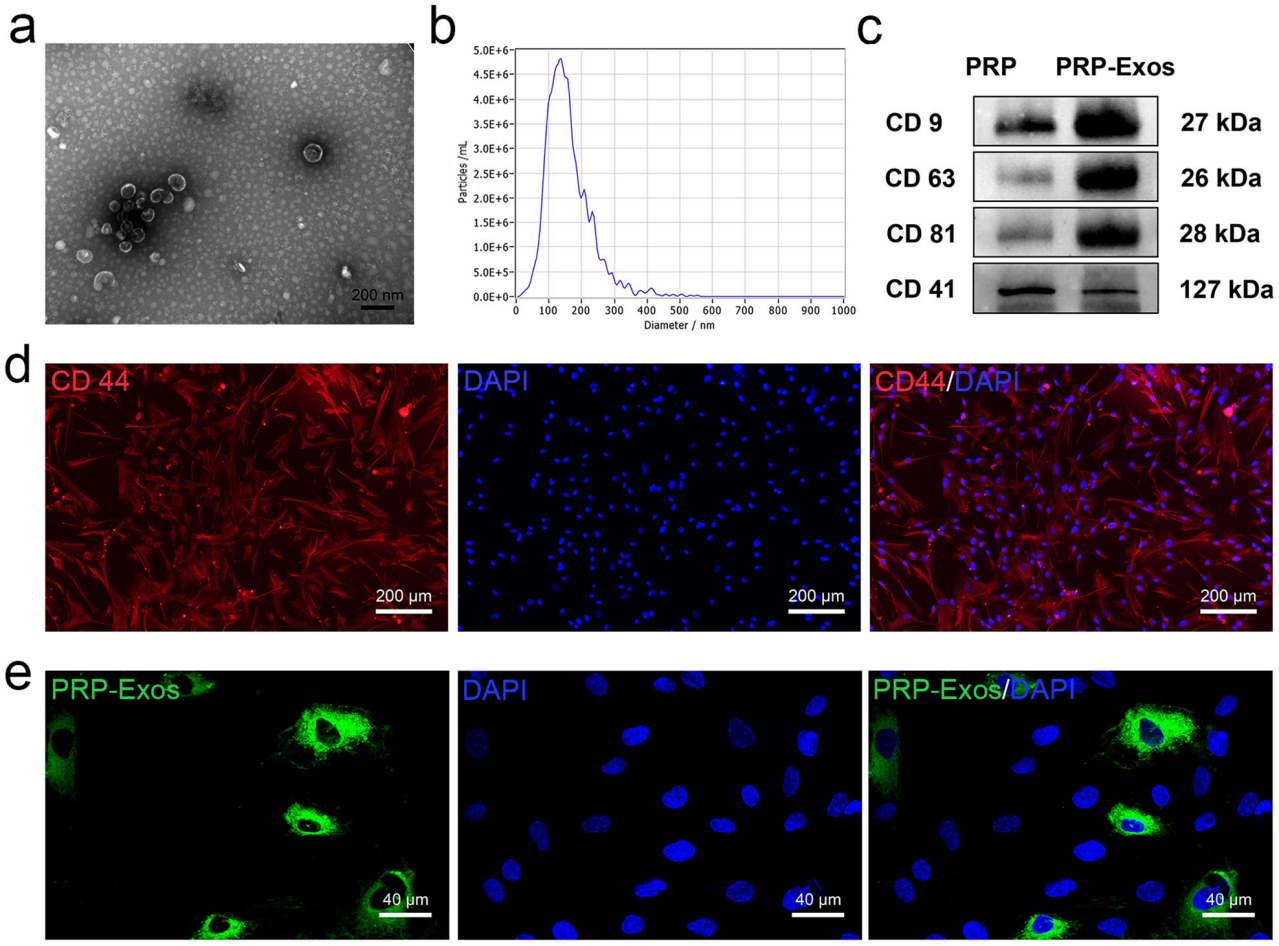
Characterization and identification of platelet-rich plasma-derived exosomes (PRP-Exos) and tendon stem/progenitor cells (TSPCs). **a** PRP-Exos under transmission electron microscopy scanning has a typical “tea tray” morphology. Scale bar = 200 nm. **b** Nanoparticle tracking analysis detected the average particle size of exosomes. **c** Western blot identified platelet-rich plasma (PRP) and PRP-Exos surface signature molecules (CD9, CD63, and CD81) and platelet source signature molecules (CD41). **d** Identification of characteristic expression of TSPCs by immunofluorescence staining. Scale bar = 200 μm. **e** PKH67 staining to identify the endocytosis of PRP-Exos by TSPCs. Scale bar = 40 μm.

Using transmission electron microscopy, we characterized the isolated PRP-Exos as uniformly spherical vesicles with the characteristic “saucer-like” exosomal structure (Fig. 1a). Nanoparticle tracking analysis (NTA) revealed the average diameter of PRP-Exos to be 132.9 nm (Fig. 1b). Western blot analysis validated the expression of exosome-specific markers CD9, CD63, and CD81, along with platelet-derived markers CD41, in the exosomes (Fig. 1c). Immunofluorescence staining demonstrated a high expression of CD44 in the isolated tendon stem/progenitor cells (TSPCs), consistent with TSPC characteristics (Fig. 1d). Furthermore, PKH67-labeled green fluorescent PRP-Exos were observed to be efficiently internalized by TSPCs (Fig. 1e).

### Effect of PRP-Exos on the biological behavior of TSPCs

**Fig. 2:**
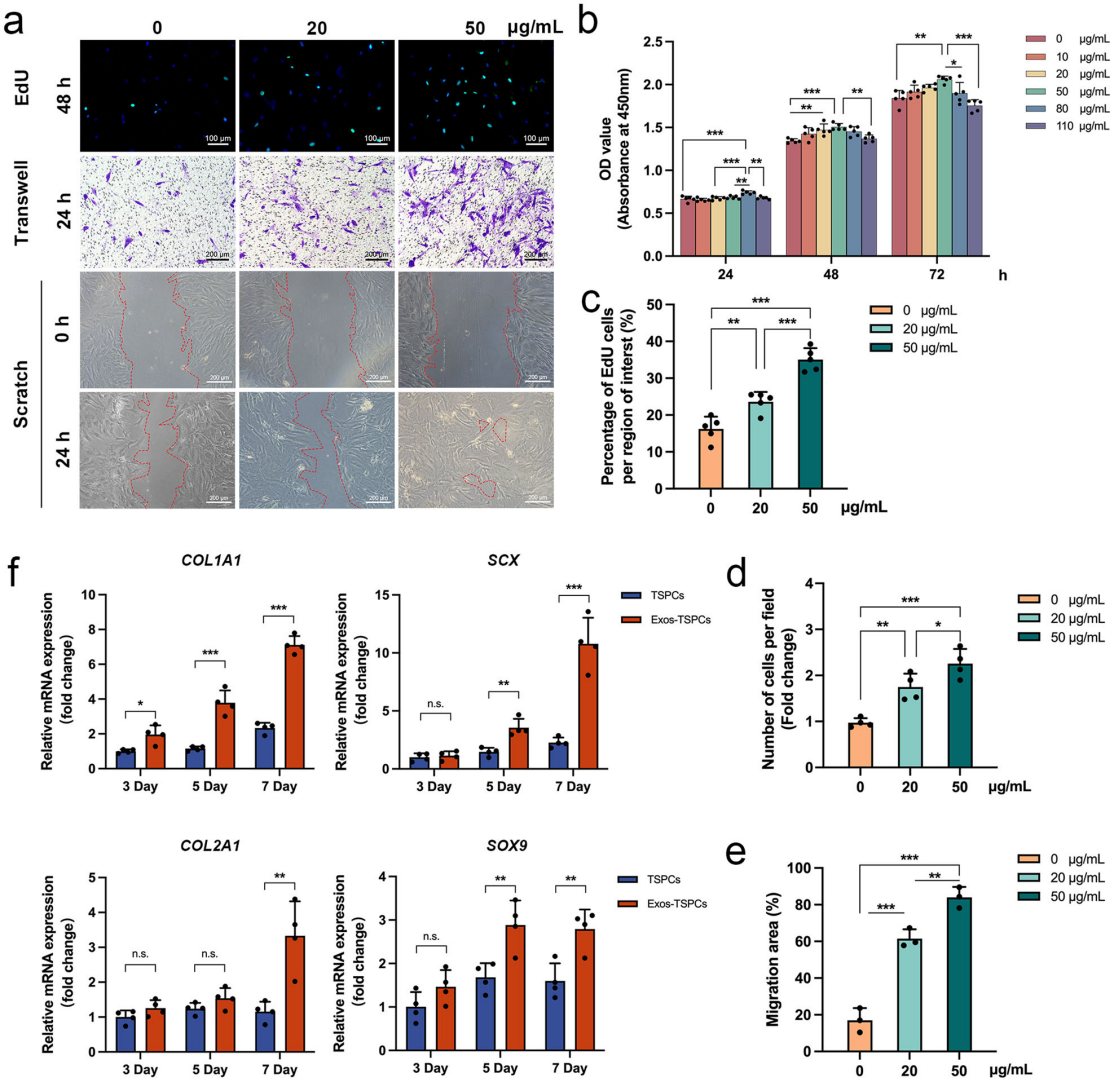
Effects of platelet-rich plasma-derived exosomes (PRP-Exos) on biological characteristics of tendon stem/progenitor cells (TSPCs). **a** The effects of PRP-Exos on TSPCs proliferation and migration were evaluated by 5-ethynyl-2’-deoxyuridine (EdU) staining, Transwell and Scratch wound healing. Scale bar = 100 μm and 200 μm. **b** The effects of PRP-Exos on TSPCs proliferation were analyzed by cell counting kit-8 (CCK-8) assay (*n* = 5). **c**–**e** Statistical analysis results of EdU staining (*n* = 5), Transwell (*n* = 4) and Scratch wound healing (*n* = 3). **f** Quantitative real-time polymerase chain reaction (qRT-PCR) showed that TSPCs and PRP-Exos-stimulated TSPCs (Exos-TSPCs) mRNA expression levels of *COL1A1*, *SCX*, *COL2A1,* and *SOX9* at 3, 5, and 7 days (Each group, *n* = 4). n.s.*P* > 0.05, *P < 0.05, **P < 0.01, ***P < 0.001. All the error bars above denote standard deviation. One-way analysis of variance with Tukey’s multiple-comparison test in (**b**–**e**) and two-tailed unpaired Student’s *t* test in (**f**).

The effect of PRP-Exos on TSPCs proliferation was assessed using the cell counting kit-8 (CCK-8) assay across various concentrations (0, 10, 20, 50, 80, and 110 μg/mL) to determine the optimal stimulation concentration. After 24 h of PRP-Exos stimulation, the 80 μg/mL group exhibited a marked increase in proliferation. At 48 and 72 h, the 50 μg/mL group demonstrated the highest proliferative capacity (Fig. 2b). Hence, PRP-Exos concentrations of 0, 20, and 50 μg/mL were selected for subsequent experiments.

The 48-h 5-ethynyl-2’-deoxyuridine (EdU) staining assay provided additional evidence, revealing a significant increase in the number of EdU-positive proliferating cells in the 50 μg/mL group compared to the 20 μg/mL group. These results aligned with the CCK-8 assay results (*P* < 0.001; Fig. 2a, c). To assess the migratory potential induced by different concentrations of PRP-Exos, we employed both the transwell and scratch wound-healing assays. The 24-h transwell assay showed that both the 20 and 50 μg/mL groups exhibited higher relative migration rates compared to the 0 μg/mL group (*P* < 0.01, *P* < 0.001). The 50 μg/mL group demonstrated superior performance compared to the 20 μg/mL group (*P* < 0.05). A similar trend was observed in the scratch wound-healing assay (Fig. 2a, d, e).

A quantitative RT-PCR (qRT-PCR) analysis was performed to assess the mRNA expression levels of tendon and cartilage markers (*COL1A1*, *SCX*, *COL2A1*, and *SOX9*) in TSPCs cultured with PRP-Exos for varying durations (3, 5, and 7 days) (Fig. 2f). Over time, a notable increase in the mRNA levels of these markers was observed. This elevation was more pronounced in the case of tendon-related markers as opposed to cartilage-related markers. Notably, a statistically significant difference in *COL1A1* mRNA expression in TSPCs cultured with PRP-Exos was observed at the 3-day time point (*P* < 0.05). Furthermore, compared to the TSPCs, TSPCs cultured with PRP-Exos exhibited significant differences in *SOX9* and *COL2A1* expression on days 5 and 7, respectively (*P* < 0.01). These findings suggest that PRP-Exos have the potential to enhance tendon and cartilage differentiation in TSPCs in vitro, thereby indicating their promise in the treatment of RCT.

### Dynamics of the FG-PRP-Exos sustained-release system

Due to the challenges in localizing the PRP-Exos solution in the rabbit rotator cuff region, we employed FG to enhance the retention and stability of exosomes.

**Fig. 3:**
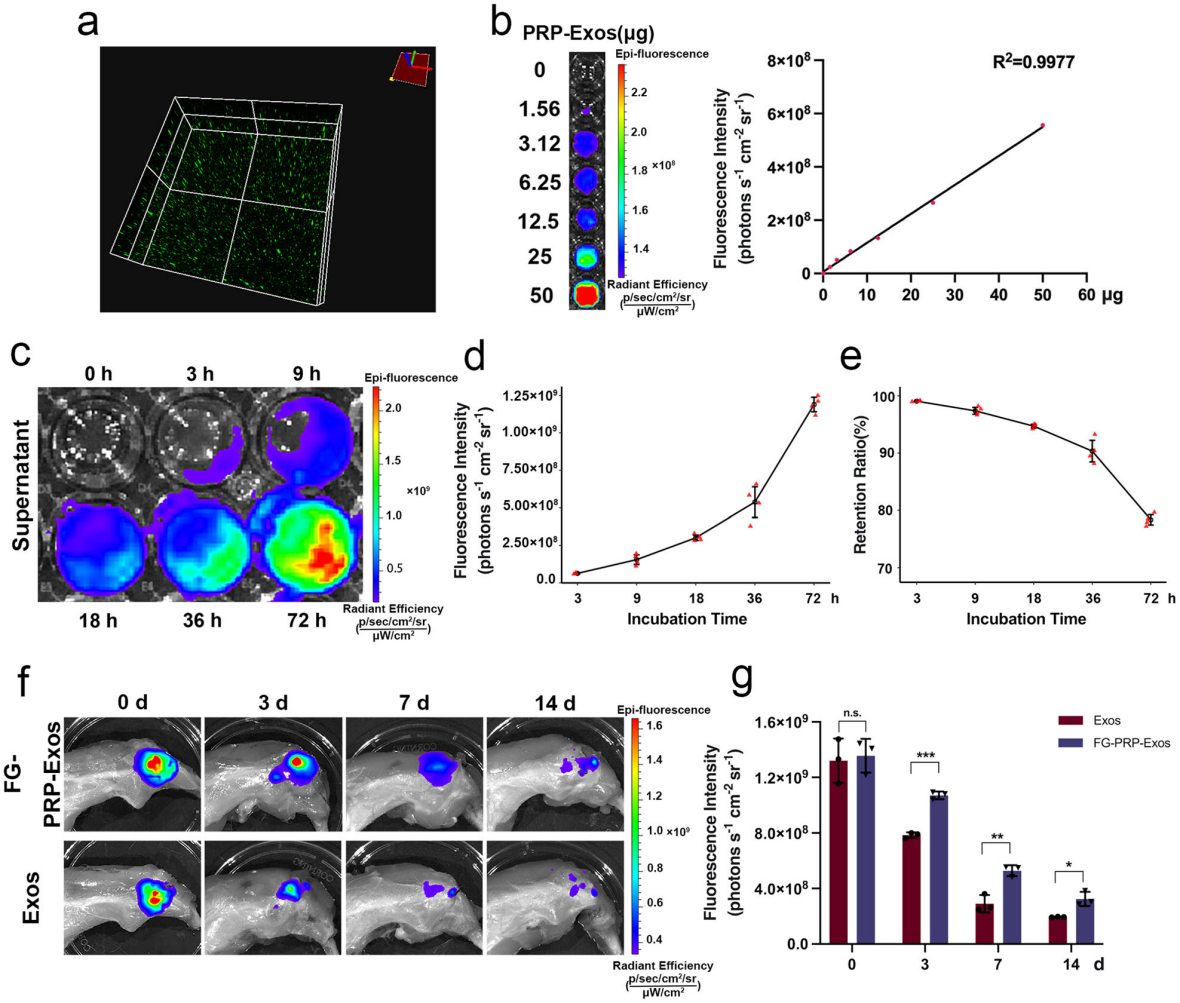
Dynamic analysis of fibrin gel-platelet-rich plasma-derived exosomes (FG-PRP-Exos) sustained-release system and determination of gel retention capacity at rotator cuff bone-tendon interface (BTI). **a** Platelet-rich plasma-derived exosomes (PRP-Exos) distribution within fibrin gel (FG). **b** Standard curve of fluorescence intensity and exosome content (*R*^2^ = 0.9977). **c** The IVIS Lumina imaging system was used to detect the sustained release of PRP-Exos embedded in FG into phosphate-buffered saline (PBS) within 0, 3, 9, 18, 36, 72 h. **d**, **e** The fluorescence intensity curve and the retention rate of FG-PRP-Exos at different incubation times, calculated according to the standard curve (both *n* = 4). **f** At 0, 3, 7, 14 d, the retention of PRP-Exos encapsulated in FG-PRP-Exos or PBS solution at the site of rotator cuff injury. **g** Fluorescence signal retained by PRP-Exos at each time point (*n* = 3). n.s.*P* > 0.05, *P < 0.05, **P < 0.01, ***P < 0.001. All the error bars denote standard deviation. Statistical method, two-tailed unpaired Student’s *t* test in (**g**).

To evaluate the dispersion of PRP-Exos in FG, we observed PKH67-labeled exosomes under a fluorescence microscope, which revealed a uniform distribution of PRP-Exos (Fig. 3a). In vitro detection results, obtained using the IVIS Lumina Imaging System, demonstrated a linear correlation between fluorescence signal intensity and the quantity of DiR-labeled exosomes, yielding an *R*^2^ value of 0.9977 (Fig. 3b). FG-PRP-Exos samples were collected at various time intervals (Fig. 3c), and their quantity was found to increase with prolonged incubation. This observation suggests a gradual increase in the release of PRP-Exos from the FG (Fig. 3d). After 72 h, a total of 108.27 μg of exosomes were released. Initially, each FG-PRP-Exos sample contained 500 μg of exosomes, enabling us to calculate the retention rate of PRP-Exos in the FG (Fig. 3e). At the 72-h mark, FG-PRP-Exos still retained 78.35% of the exosomes.

We further validated the in vivo retention capacity of FG-PRP-Exos at the injury site. The data revealed detectable fluorescence signal intensities in Exos group and FG-PRP-Exos group at the 14-day time point (Fig. 3f). Compared with the Exos group, the PRP-Exos group exhibited higher signal strength and a propensity for clustering at all time intervals. In addition, the analysis of signal intensity at different time points indicated that FG had the most pronounced impact on the retention of PRP-Exos on day 3 post surgery (*P* < 0.001), and on days 7 and 14 post surgery, it significantly enhanced the in vivo stability of exosomes (*P* < 0.01, *P* < 0.05; Fig. 3g).

### Biomechanical assessment

**Fig. 4:**
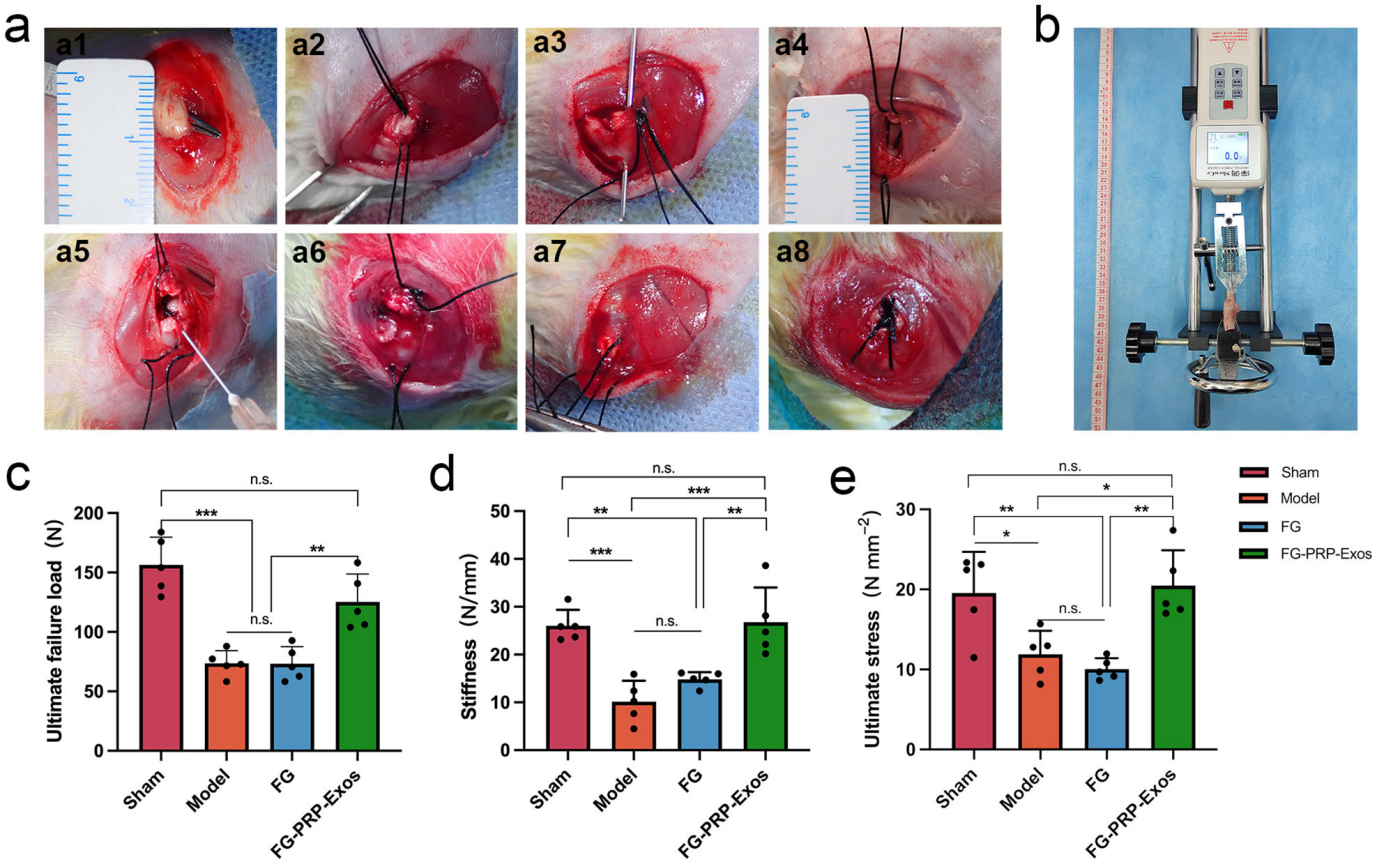
Surgical procedure and biomechanical testing for rotator cuff tear repair. **a1** The infraspinatus tendon was carefully bluntly dissected. **a2** Two 3-0 Ethibond sutures are passed through the tendon. **a3** The insertion point of the infraspinatus tendon was cut, and a 5 mL syringe needle was used to construct double rows of parallel bony tunnels at the greater tuberosity of the humerus. **a4** Two sutures were passed through the bone tunnel from the inside to the outside. **a5** Different treatment modalities were administered according to the protocol using a double syringe. **a6** Fibrin gel-platelet-rich plasma-derived exosomes (FG-PRP-Exos) gelled in situ at the bone-tendon interface. **a7** Cover the infraspinatus tendon completely with gel. **a8** Gross observation after modified Mason–Allen suture. **b** The biomechanical testing instrument showed that the humerus was stably fixed at the lower end, and the infraspinatus tendon was fixed on the attachment through the clamp, and failed under the final compliance. **c**–**e** Ultimate failure load, stiffness, and ultimate stress at 12 W after surgery (Sample type are denoted below each bar, *n* = 5). n.s.*P* > 0.05, *P < 0.05, **P < 0.01, ***P < 0.001. Error bars are defined as standard deviation. Statistical method, One-way analysis of variance with Tukey’s multiple-comparison test in (**c**–**e**).

The biomechanical assessment conducted at 12 weeks post surgery (Fig. 4b) revealed no statistically significant differences between the Sham group and the FG-PRP-Exos group, as well as between the Model group and the FG group. The FG-PRP-Exos group exhibited a significantly higher ultimate failure load of 125.2 ± 23.49 N, in contrast to the Model group (73.56 ± 10.69 N) and the FG group (73.32 ± 14.18 N), demonstrating a statistically significant difference (*P* < 0.01; Fig. 4c). Similarly, stiffness and ultimate stress were comparable. The FG-PRP-Exos group (26.76 ± 7.25 N/mm) demonstrated significantly higher stiffness values compared to the Model group (10.13 ± 4.37 N/mm) and the FG group (14.83 ± 1.50 N/mm), with a notable statistical difference (*P* < 0.001, *P* < 0.01; Fig. 4d). In the case of ultimate stress, the FG-PRP-Exos group exhibited significant differences compared to the Model and FG groups (*P* < 0.05, *P* < 0.01), indicative of a favorable recovery outcome (Fig. 4e).

### Ultrasonography assessment of target muscle recovery

**Fig. 5:**
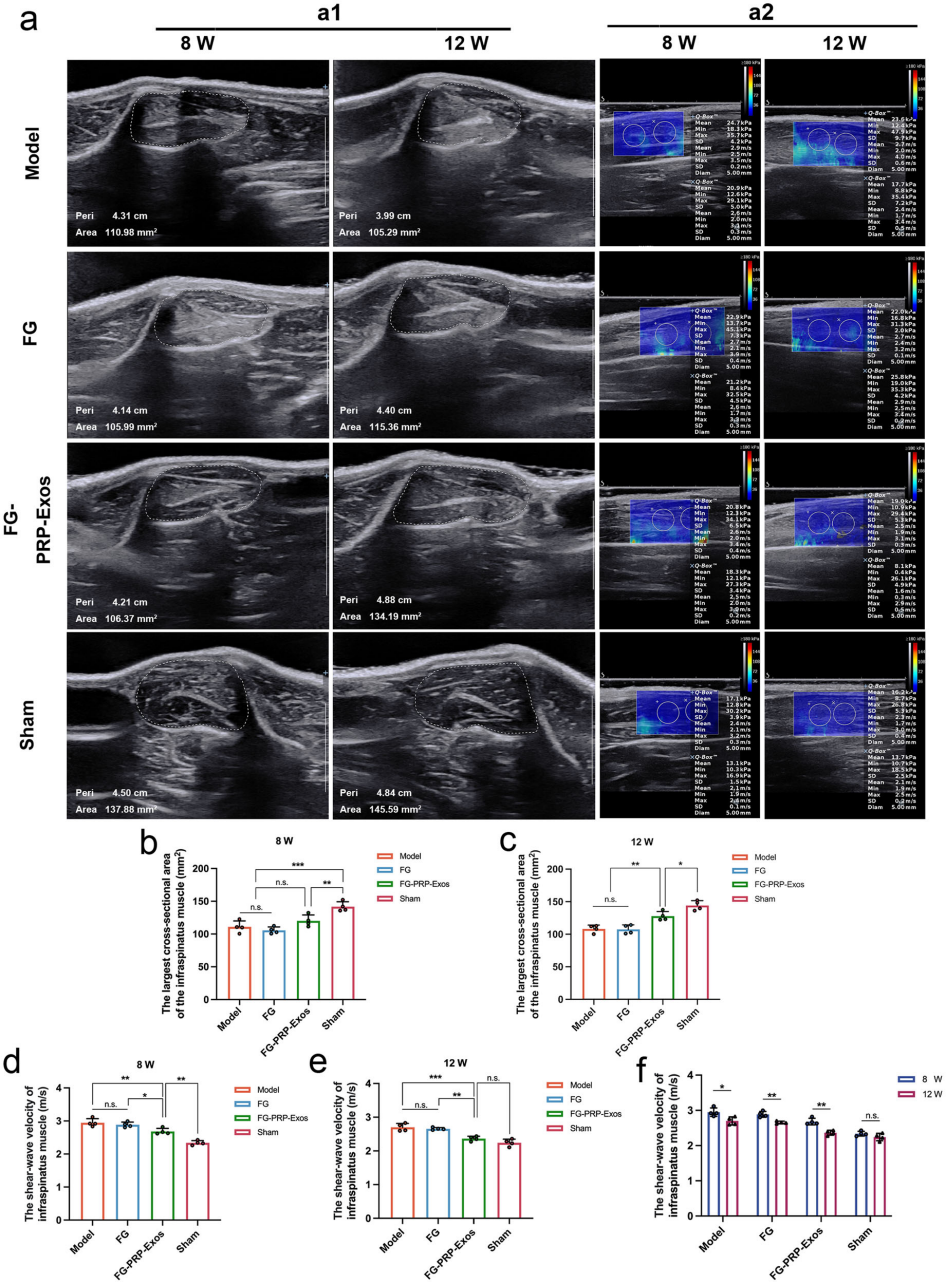
Ultrasound images of the 8 W and 12 W surgical lateral target muscles of the infraspinatus muscle. **a1** Conventional ultrasound measures the muscle dimension of the largest cross-sectional area of the infraspinatus muscle, with the white dashed line showing the region of interest (ROI). **a2** Shear wave elastography (SWE) image of infraspinatus muscle, ROI diameter = 5 mm. Statistical analysis was performed by calculating the maximum diameter cross-sectional area (**b**, **c**) and shear wave velocity value (**d**, **e**) of the target muscles (Each group, *n* = 4). **f** Comparison of 8 W to 12 W dynamic target muscle recovery in each treatment group (Each group, *n* = 4). n.s. *P* > 0.05, *P < 0.05, **P < 0.01, ***P < 0.001. All the error bars above denote standard deviation. Statistical methods, One-way analysis of variance with Tukey’s multiple-comparison test in (**b**–**e**) and two-tailed unpaired Student’s *t* test in (**f**).

**Fig. 6:**
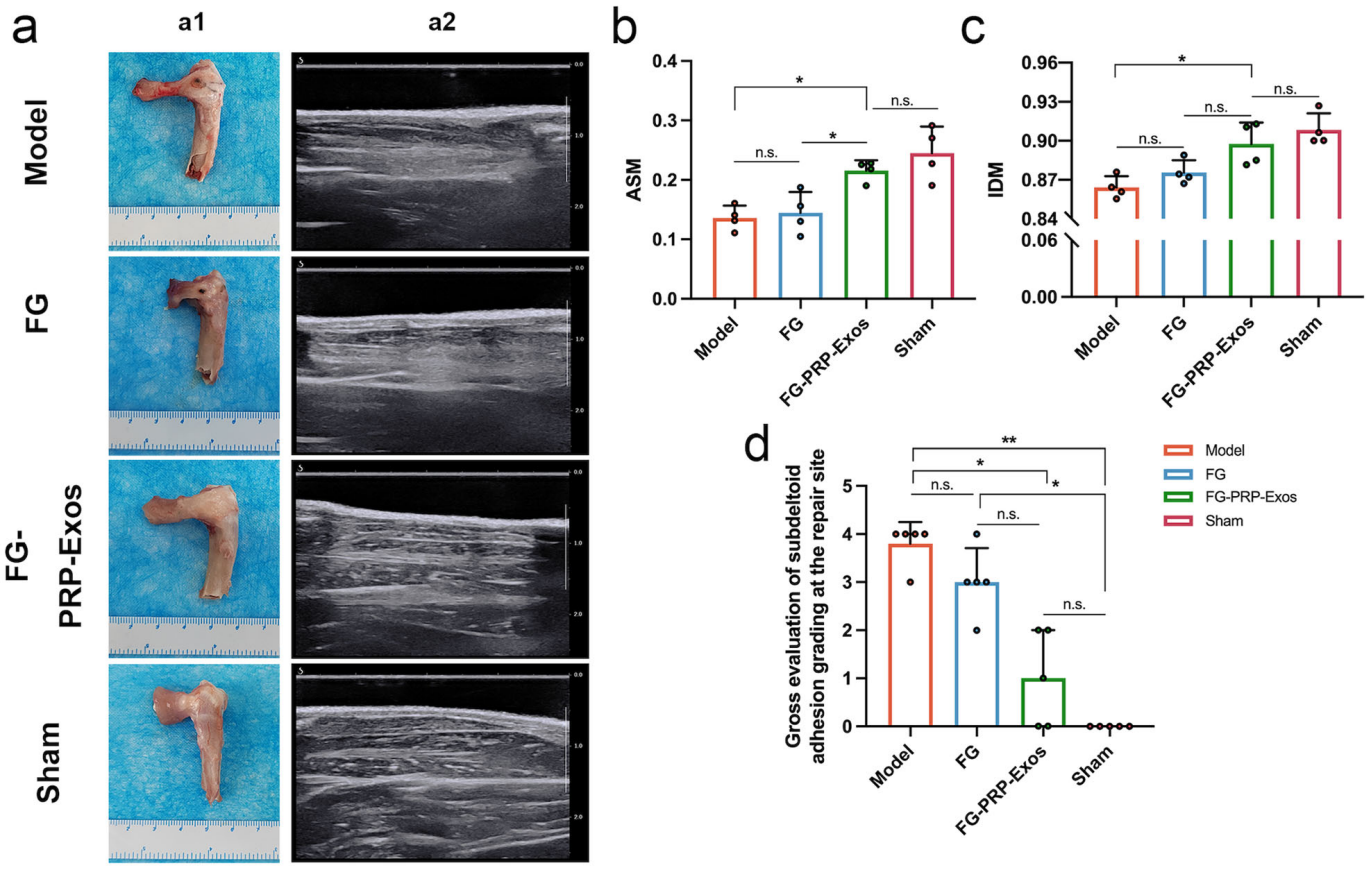
Gross image of the rotator cuff and ultrasound image of target muscles at 12 W. **a1** Gross image of the rotator cuff injury area at 12 W. **a2** Texture on each longitudinal section of the infraspinatus muscle. **b**, **c** Texture analysis parameters angular second moment (ASM) and inverse difference moment (IDM) of the infraspinatus muscle at 12 W (*n* = 4). **d** Overall score of degree of subdeltoid adhesion at the repair site at 12 W (*n* = 5). n.s. *P* > 0.05, *P < 0.05, **P < 0.01. All the error bars above are defined as standard deviation. Statistical methods, One-way analysis of variance with Tukey’s multiple-comparison test in (**b**, **c**) and Kruskal–Wallis test in (**d**).

Conventional ultrasound assessment of the target muscle: Conventional ultrasound assessments of the infraspinatus muscle revealed varying degrees of atrophy, characterized by a reduction in maximum cross-sectional area, increased echogenicity, and a blurred muscle texture (Figs. 5 and 6). At 8 weeks, no significant differences in the maximum cross-sectional area were observed among the Model, FG, and FG-PRP-Exos groups (Fig. 5b). However, at 12 weeks, the FG-PRP-Exos group exhibited a higher cross-sectional area compared to the Model and FG groups (*P* < 0.01), indicating less muscle atrophy and a lower statistical difference from the Sham group (*P* < 0.05; Fig. 5c).

Shear wave elastography (SWE) ultrasound assessment of infraspinatus muscle stiffness: SWE showed that, at 8 weeks post surgery, the FG-PRP-Exos group exhibited a lower shear wave velocity compared to both the Model group and the FG group (*P* < 0.01, *P* < 0.05), ranking second only to the Sham group (*P* < 0.01; Fig. 5d). By the 12-week mark, the FG-PRP-Exos group demonstrated the lowest shear wave velocity, with no statistically significant difference compared to the Sham group, indicative of superior muscle hardness (Fig. 5e). To assess the self-recovery effects across various treatment groups, we analyzed the changes in shear wave velocity in the infraspinatus muscle at various time points. From 8 to 12 weeks of recovery time, the FG-PRP-Exos and FG groups exhibited the most significant reduction in muscle stiffness (*P* < 0.01), followed by the Model group (*P* < 0.05), while the Sham group displayed no significant difference (Fig. 5f).

Conventional ultrasound for quantitative assessment of muscle texture: At 8 and 12 weeks post surgery, we macroscopically observed the regenerative status of the BTI. All repair groups exhibited tendon thinning, varying degrees of adhesion, and proliferation. The Model and FG groups exhibited suture nodes, whereas the FG-PRP-Exos group demonstrated a thicker tendon, with the nodes encapsulated and the muscle showing a slightly greater volume (Fig. 6a1). At 12 weeks, there was no statistically significant difference in the angular second moment (ASM) between the Model group and the FG group, but it was lower than that observed in the FG-PRP-Exos group (both *P* < 0.05). Moreover, the FG-PRP-Exos group exhibited a higher inverse difference moment (IDM, *P* < 0.05) compared to the Model group. Elevated ASM and IDM values are indicative of enhanced muscle texture uniformity, which in turn may reflect a reduced extent of fat infiltration and fibrosis (Fig. 6a2–c)^25^.

### Gross evaluation of subdeltoid adhesion grading at the repair site

No tendon ruptures were observed at the 12-week post-surgery mark. Both the Model group and FG group exhibited more pronounced fibrotic scar formation, coupled with a higher degree of adhesion to the surrounding tissues, rendering separation challenging. In contrast, the FG-PRP-Exos group exhibited the mildest proliferation and adhesion around the rotator cuff, allowing for easy separation through gentle traction. The degree of subdeltoid adhesion in the FG-PRP-Exos group was significantly lower compared to that in the Model group (*P* < 0.05), with no significant difference between the Model and FG groups (Fig. 6d).

### Micro-computed tomography (Micro-CT)

**Fig. 7:**
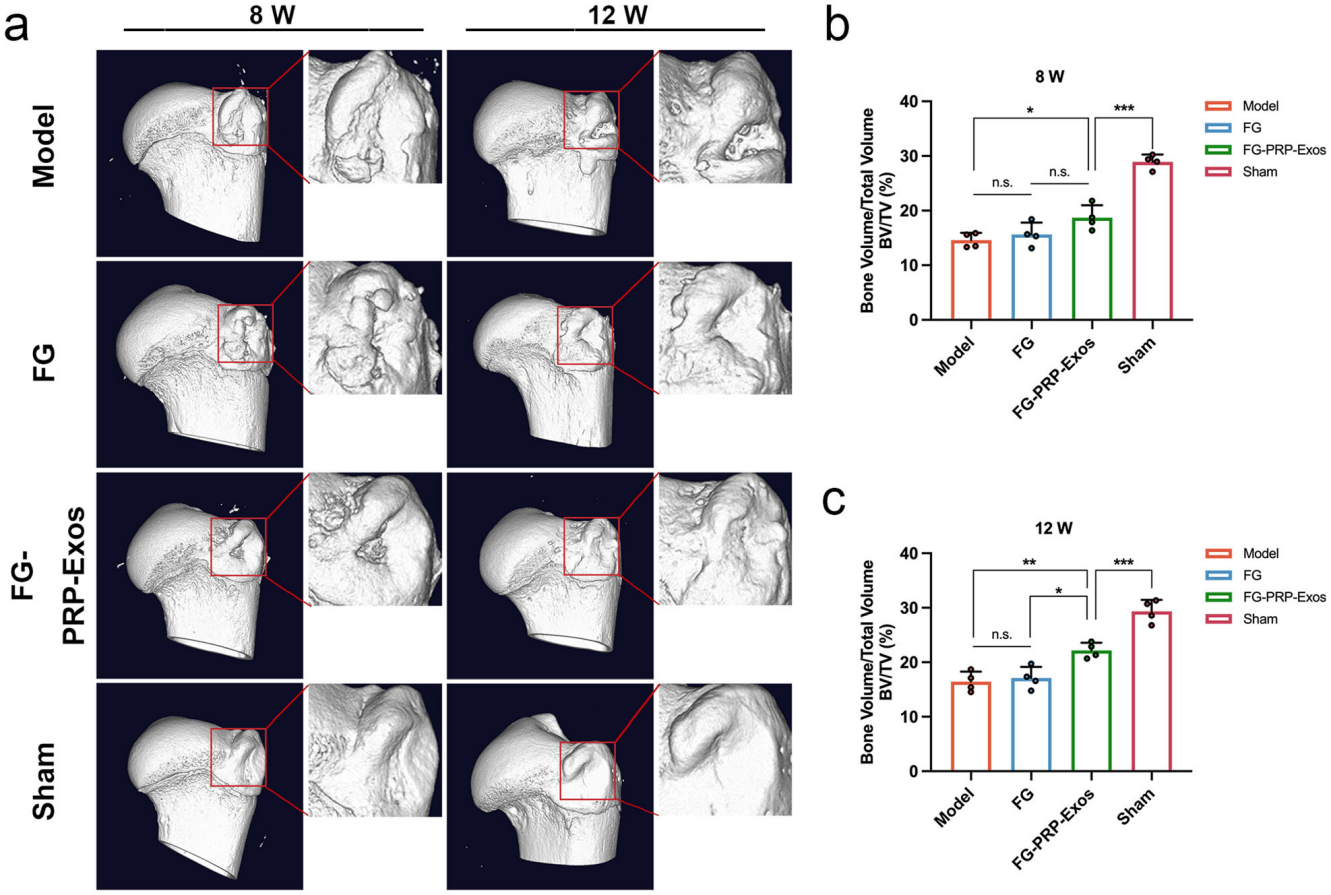
Micro-computed tomography (micro-CT) scan results. **a** Micro-CT three-dimensional (3D) reconstruction image of the humeral head region on the treatment side, red range was the region of interest (ROI) included in the assessment, ROI = 5 mm × 5 mm^2^. **b**, **c** ROI region bone volume/total volume (BV/TV) analysis results (Each group, *n* = 4). n.s. *P* > 0.05, *P < 0.05, **P < 0.01, ***P < 0.001. Error bars denote standard deviation. One-way analysis of variance with Tukey’s multiple-comparison test in (**b**, **c**).

Micro-CT scanning and three-dimensional (3D) reconstruction revealed significant new bone regeneration and remodeling in the FG-PRP-Exos group at 8 and 12 weeks post surgery. This enhancement was notably superior to both the Model and FG groups. In contrast, the reconstruction results of the Model and FG groups revealed significant non-healing bone defects at the greater tuberosity of the humerus, characterized by poor surface continuity and ectopic ossification. The surface continuity of the greater tuberosity in the FG-PRP-Exos group improved at the 12-week time point compared to the 8-week time point, resembling the structure and morphology observed in the Sham group (Fig. 7a). At 8 weeks, the bone volume/total volume (BV/TV) of the FG-PRP-Exos group was slightly higher than that of the Model group (*P* < 0.05), but there was no significant difference between the FG group (Fig. 7b). Furthermore, the BV/TV in the FG-PRP-Exos group displayed significant regenerative advantages compared to the other two groups at the 12-week time point (*P* < 0.01, *P* < 0.05; Fig. 7c).

### Histological analysis

**Fig. 8:**
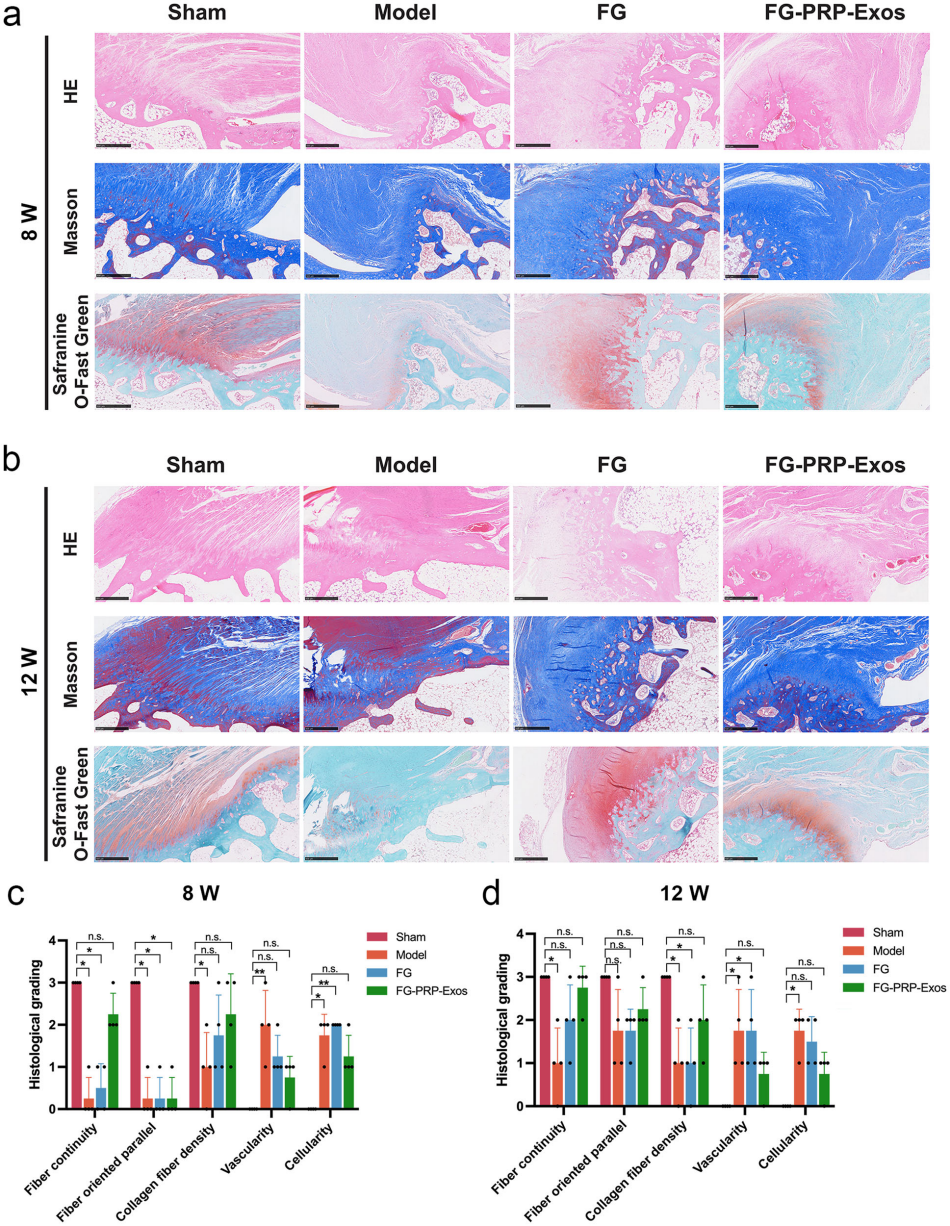
At 8 W and 12 W histological staining and grading. **a**, **b** Hematoxylin–eosin staining (HE), Masson staining, Safranine O-Fast Green staining results. Scale bar = 500 μm. **c**, **d** Statistical results of histological analysis of each group (*n* = 4). n.s. *P* > 0.05, *P < 0.05. All the error bars above denote standard deviation. Statistical methods, Kruskal–Wallis test in (**c**, **d**).

During the post-surgery recovery period spanning from 8 to 12 weeks, the FG-PRP-Exos group demonstrated a superior BTI characterized by a more organized arrangement of collagen fibers with increased continuity. This group also exhibited minimal infiltration of inflammatory cells and vascularization, resembling the natural structure of BTI. Notably, at the 12-week mark, the FG-PRP-Exos group exhibited the formation of cartilage pits and a state resembling natural interposition. Conversely, the Model and FG groups both displayed loosely arranged collagen fibers resembling scar-like tissue regeneration, indicative of inadequate BTI repair. Furthermore, compared to the Model group, the FG and FG-PRP-Exos groups exhibited a higher presence of eosinophilic substances, which may constitute the primary components of the cartilage matrix. Among these groups, the FG-PRP-Exos group exhibited a more pronounced cartilage morphology, likely attributed to the PRP-Exos (Fig. 8a, b).

The histological scores at 8 weeks demonstrated that, compared to the Sham group, the FG-PRP-Exos group had no significant differences in regenerative fiber continuity, density, vascularity, and cellularity. In addition, the Model group exhibited less than ideal repair outcomes, with pronounced vascularization (*P* < 0.01). By the 12th week, the FG-PRP-Exos group exhibited enhanced collagen fiber regeneration in contrast to the Model and FG groups (Fig. 8c, d).

## Discussion

This study is the first to evaluate the efficacy to FG-encapsulated PRP-Exos in the context of RCT. Through multi-modality imaging examinations, including conventional ultrasound, SWE ultrasound, and micro-CT, coupled with histological staining, we jointly assessed the effect of PRP-Exos on the enhancement of rotator cuff regeneration. Our findings indicate significant improvement in the post-injury healing process of the rotator cuff resulting from this combinatory approach.

Previous studies have primarily centered on utilizing stem cell-derived exosomes for the repair of RCT^26^. However, only a few scholars have explored the potential of PRP-Exos in rotator cuff repair. Moreover, few studies have investigated the initial use of FG to facilitate in situ gelation of PRP-Exos during surgery, followed by suturing the tendon to the humeral tuberosity post-implantation. Notably, the intricate anatomical structure^27^ and the irregular shape of the cuff after tearing raise questions about the feasibility and therapeutic efficacy of PRP-Exos, which became the key focus of this study. In this study, we initially screened an optimal concentration of 50 μg/mL PRP-Exos, which demonstrated the highest potential for inducing significant phenotypic changes and lineage differentiation in TSPCs. Subsequently, we validated that FG-encapsulated PRP-Exos can enhance the biomechanical properties of the rotator cuff, facilitate remodeling of the BTI, and contribute to the recovery of the target muscle in vivo. A multi-modality evaluation of the treatment’s effectiveness showed that the utilization of FG-PRP-Exos yields positive outcomes for rotator cuff remodeling.

We demonstrated the ability of PRP-Exos to regulate positive remodeling of the rotator cuff following injury. Recent studies have demonstrated the potential benefits of exosome therapy in rotator cuff repair, albeit with complex underlying mechanisms that remain unclear. Unlike stem cell transplantation, which carries potential risks of tumorigenesis and immunorejection^28^, exosome therapy is considered safe. Exosomes sourced from various origins enhance the rotator cuff repair process through multiple pathways and targets, including the regulation of inflammatory responses^29^, and the promotion of fibrocartilage regeneration^30^. These findings offer promising therapeutic avenues. However, challenges associated with production, regulatory considerations, clinical applications, and associated costs, including equipment, personnel, and quality control, may limit their clinical translation. PRP-Exos are derived from PRP and contain a rich array of active growth factors, such as platelet-derived growth factor, vascular endothelial growth factor, and fibroblast growth factor^16,17^. These growth factors theoretically play pivotal roles in regulating the positive remodeling of the BTI following acute RCT. To the best of our knowledge, no prior studies have investigated the reparative effects of PRP-Exos in acute RCT. However, in previous studies, Zhang et al. effectively promoted cartilage protection in subtalar osteoarthritis using PRP-Exos^31^ and Lyer et al. validated their ability to accelerate functional recovery after muscle injuries^32^. These findings could be instrumental in the comprehension of rotator cuff remodeling. In this study, we first explored the effects of PRP-Exos on TSPCs. We subsequently identified an optimal drug concentration of 50 µg/mL for PRP-Exos, which effectively induced significant phenotypic changes in TSPCs in vitro. As expected, PRP-Exos influenced the differentiation of TSPCs toward tendon and cartilage lineages. qRT-PCR revealed a significant increase in the expression levels of genes such as *COL1A1*, *SCX*, *COL2A1*, and *SOX9* on days 5 and 7. These findings provide a solid foundation for further investigation into the in vivo treatment of RCT with PRP-Exos.

In this study, we selected FG as the carrier for PRP-Exos due to its established biosafety and ease of clinical acquisition. Previous research has demonstrated the efficacy of FG-encapsulated exosomes in various applications, such as spinal cord injury repair^33^, tendon regeneration^34^, incisional hernia healing^35^, ischemic wound healing^36^, showcasing notable therapeutic effects. Building upon this evidence, our study delved further into investigating the appropriate implantation methods for FG and PRP-Exos. The implantation technique for combining FG and PRP-Exos also necessitates careful consideration. One commonly employed approach involves implantation following ex vivo gelation. Ren et al. inserted a solid gel, composed of purified exosome product and TISSEEL, into a rat RCT, thereby expediting the healing process in a rat RCT model^24^. Another approach is in situ gelation during surgery, wherein the timing of gelation in relation to tendon suturing is a key factor. Lu et al. encapsulated the adipose-derived stromal vascular fraction in FG and injected it into the vicinity of the rotator cuff tissue after tendon suturing, resulting in significant restoration of biomechanical properties^37^. Few investigations have ventured into in situ gelation prior to tendon suturing due to the uneven structural nature of the tear site. However, we found that during surgery, FG could remain in place at the BTI after 5–8 s of gelation, although it may not be as ideally suited for in situ injection as a closed cavity. The gel firmly adhered to the BTI, effectively sealing and binding the torn tendon. We anticipate that FG will complement PRP-Exos in mitigating inflammation and reducing postoperative bleeding owing to its anti-inflammatory and hemostatic properties.

We employed multi-modality imaging to assess the effectiveness of the combination of PRP-Exos and FG in RCT repair. The assessment of target muscle recovery is crucial in evaluating the regeneration of the rotator cuff. Utilizing conventional ultrasound for evaluating the extent of target muscle recovery, we observed significant inhibition of muscle atrophy treated with FG-PRP-Exos, accompanied by larger muscle dimensions. Additionally, the presence of clearer echo signals in the texture indicated reduced fat infiltration^10^. Furthermore, we utilized SWE ultrasound to detect and quantitatively analyze target muscle stiffness. The FG-PRP-Exos group exhibited lower stiffness compared to the FG and Model groups. Elevated stiffness levels are indicative of lower muscle quality and increased fibrosis, underscoring the efficacy of SWE ultrasound in assessing post-RCT recovery. In addition, 3D reconstructions obtained through micro-CT indicated complete bone healing at the site of the rotator cuff injury, with the continuity and morphological integrity of the bone surface closely resembling the normal structure. These findings were further corroborated by histological staining results. A multi-modality evaluation is necessary, providing a more comprehensive and dependable dataset for post-RCT recovery evaluation. Our study is the first to demonstrate the significant therapeutic effect of FG-PRP-Exos in the acute repair of RCT, thus laying the groundwork for further elucidation of its mechanism of action.

The potential advantages of combining FG and PRP-Exos are also evident in their utility for clinical translation. FG is readily available and easily obtainable in clinical settings^38^. PRP-Exos offer clinical translation benefits in the context of regulatory compliance and safety considerations. Unlike the constraints faced by PRP in clinical applications, such as the lack of standardization and challenges in quality control, PRP-Exos have diverse sources. They can be prepared either autologously or on a large scale from discarded platelets during clinical-grade allogeneic transfusion processes, facilitating their application in clinical settings^17^. In contrast to PRP, which depends on an individual’s physiological condition, PRP-Exos offer a universal solution, even for patients unable to undergo PRP extraction due to a poor medical history or subpar PRP quality^16^. Notably, freeze-dried exosome products appear to be suitable for clinical use. In a previous study, a United States-patented technology (US Patent 20160324A1) was utilized to convert a purified exosome product into freeze-dried powder and package it in vials. This approach has successfully demonstrated its efficacy in promoting tendon repair in an ex vivo canine model^39^. The freeze-dried product offers stability and retains its reparative properties when stored at room temperature, presenting a novel avenue for the clinical translation of PRP-Exos.

Several potential mechanisms may explain how PRP-Exos accelerates rotator cuff repair and regeneration. These mechanisms include inducing changes in cell phenotype, promoting cell recruitment, and stimulating angiogenesis and osteogenesis. Indeed, the potential repair mechanisms of PRP-Exos largely hinge on the components within PRP. Following tissue injury, platelets, acting as the initial cells to aggregate and induce wound homeostasis remodeling, actively participate in various stages of tissue healing through multiple bio-signaling pathways^40^. Platelets release a variety of growth factors that serve as the primary contributors to regenerative efficacy, alongside cytokines and the extracellular matrix. Together, these elements regulate vascular remodeling, foster the regeneration of injured tissue, and activate endogenous cells to carry out repair functions^41^. PRP-Exos, enriched with higher levels of growth factors compared to PRP, are considered to harbor immense potential. Reports confirm that payloads like PDGF and VEGF within platelet-derived extracellular vesicles aid in inducing changes in cell phenotype, promoting cell recruitment and adhesion, and enhancing angiogenesis^42^. Furthermore, platelet-derived extracellular vesicles have been demonstrated to enhance bone differentiation and induce bone regeneration by activating the Akt/Bad/Bcl-2 pathway^22^. The injured area of the rotator cuff is characterized by a scarcity of cells, diminished vascular function, and the scar tissue lacks the natural gradient structure and orderly fiber arrangement. Improving vascular function and inhibiting scar proliferation are crucial steps in the healing process. Building upon existing research findings and advantages of PRP-Exos, it is reasonable to speculate that they may offer extensive therapeutic potential in repairing rotator cuff tear. Consequently, exploring the repair mechanism of PRP-Exos will be the next research focus for our team.

**Fig. 9:**
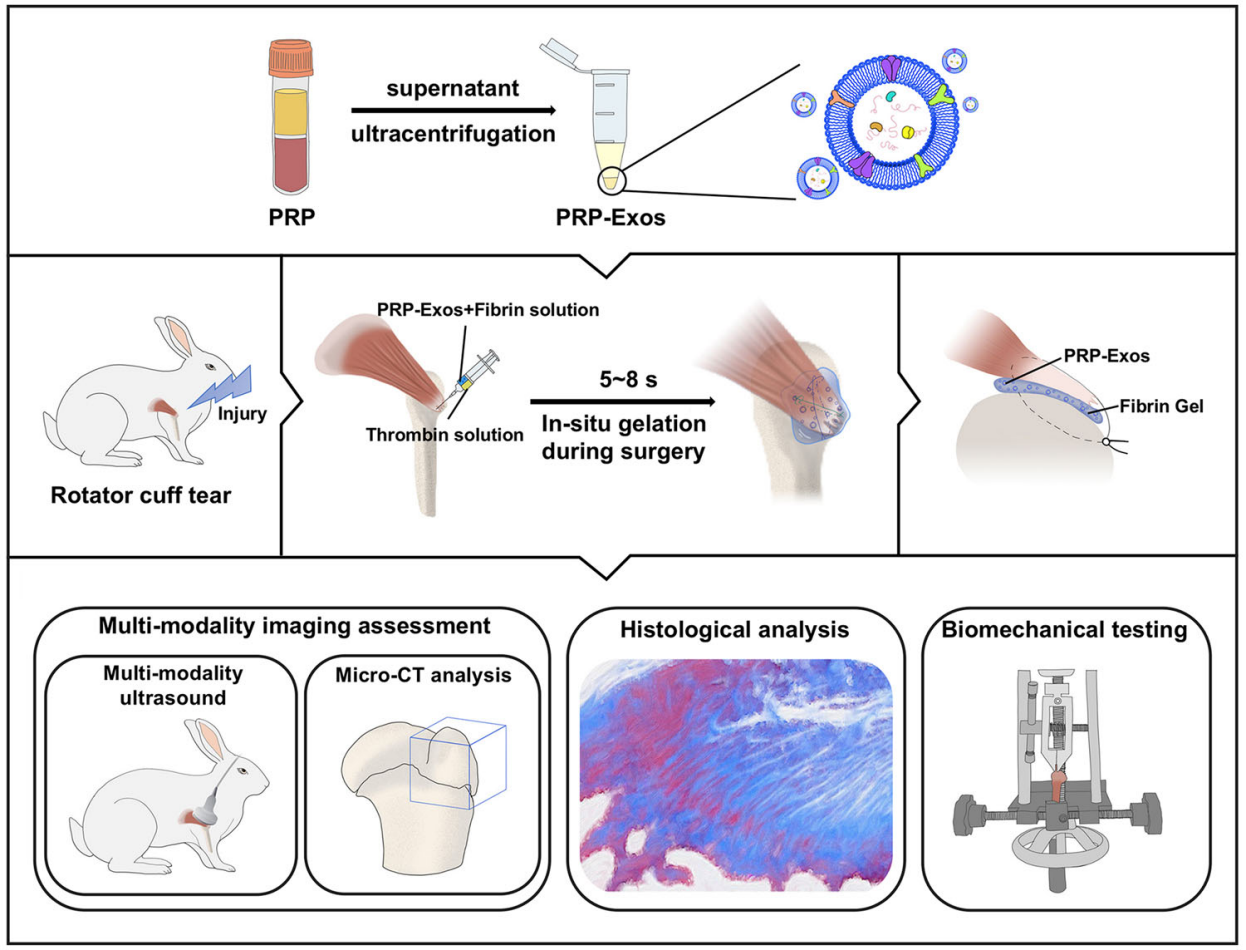
The fibrin gel (FG)-encapsulated platelet-rich plasma-derived exosomes (PRP-Exos) promoted the repair of rotator cuff tear (RCT). The FG-encapsulated PRP-Exos as a sustained-release material, effectively promoted bone-tendon interface (BTI) remodeling through in situ gelation during surgery. The efficacy of the treatment for RCT was reliably assessed using multi-modality assessment strategy.

Using PRP-Exos with the capacity for large-scale production and clinically available FG, we developed an FG-PRP-Exos sustained-release system, which facilitates in situ gelation during surgery. This study comprehensively evaluated the favorable impact of FG-PRP-Exos on the post-injury healing of the rotator cuff from multiple perspectives, encompassing multi-modality imaging, pathological findings, and biomechanical analyses (Fig. 9). Our findings suggest that the combination of PRP-Exos and FG presents a safe and dependable avenue for potential translation into clinical treatment for RCT.

Nevertheless, this study has several limitations. Although we validated the beneficial impact of PRP-Exos in facilitating RCT repair, the intricate signaling pathways and underlying repair mechanisms remain unclear, necessitating further exploration. Furthermore, we only validated the feasibility and effectiveness of the in-situ gelation implantation approach employing FG loaded with PRP-Exos. However, a comparative assessment of the advantages between this method and graft implantation following ex vivo gelation remains inconclusive.

## Methods

All procedures detailed in this study were approved by the Ethics Committee of the Chinese PLA General Hospital (No. 2023-X19–32). All experiments were performed at the State Key Laboratory of Kidney Diseases, Chinese PLA General Hospital in Beijing, China. We have complied with all relevant ethical regulations for animal use.

### Preparation of PRP

PRP was prepared following a previously reported protocol^43^. A total of 8 mL of whole blood was collected from healthy volunteers and placed in a 10-mL sterile tube containing 3.8% w/v sodium citrate. PRP was rapidly prepared using a two-step centrifugation method. After the first centrifugation (400×*g*, 10 min), the sample was separated into three layers: the top layer, which was cell-free plasma; the middle layer, rich in platelets; and the bottom layer, composed of red and white blood cells. The top and middle layers were carefully transferred to a new sterile tube for a second centrifugation (800×*g*, 10 min). The bottom third of the resulting sample was the PRP. Both the whole blood and the resulting PRP samples were forwarded to the laboratory for analysis. PRP samples with a platelet count ranging from three to six times that of whole blood were selected for subsequent experiments.

### Isolation and characterization of PRP-Exos

PRP-Exos were isolated from PRP through the combination of polymerization precipitation and ultracentrifugation. Crude PRP-Exos were initially precipitated via polymerization precipitation using ExoQuick (EXOQ5TM-1, ExoQuick Plasma prep and Exosome precipitation kit, System Biosciences, CA, USA). To enhance purity by removing impurities such as lipoproteins, we resuspended the PRP-Exos pellet in PBS and subjected it to ultracentrifugation (4 °C, 100,000×*g*, 70 min). The purified PRP-Exos pellet was subsequently stored at −80 °C.

The final size of exosome particles was determined through NTA using ZetaView (Particle Metrix, Meerbusch, Germany). Exosome morphology was characterized using transmission electron microscopy (Hitachi, H7500, Tokyo, Japan). The concentration of exosomes was quantified using a Pierce^TM^ BCA Protein Assay Kit (#23227, Thermo Scientific, Rockford, USA). The surface marker molecules of PRP-Exos were identified via western blot analysis, with primary antibodies including anti-CD9 (1:1000, 60232-1-Ig, Proteintech, USA), anti-CD63 (1:5000, 67605-1-Ig, Proteintech, USA), anti-CD81 (1:5000, 66866-1-Ig, Proteintech, USA), and anti-CD41 antibodies (1:4000, 60350-1-Ig, Proteintech, USA). All of the original western blot bands were provided in Supplementary Information (Supplementary Fig. 1).

### Isolation and identification of TSPCs

Rat TSPCs were extracted following established protocols^44^. Briefly, the Achilles tendon obtained from suckling rats was initially minced and subjected to enzymatic digestion using 0.2% type I collagenase (Sigma-Aldrich, MO, USA) (37 °C, 1 h). After passing the cells through a 70-μm filter, they were suspended in low-glucose Dulbecco’s Modified Eagle Medium (DMEM, Gibco, CA, USA) supplemented with fetal bovine serum (10% FBS, Gibco, USA) in humidity (37 °C, 5% CO_2_). To assess the purity of the isolated TSPCs, the presence of CD44 molecules on their surface was evaluated using the anti-CD44 antibody (1:100, 15675-1-AP, Proteintech, USA). TSPCs from passages three to six were used for subsequent experiments.

### Cellular internalization of PRP-Exos

PRP-Exos were labeled with PKH67 dye (Fluorescence Biotechnology, China). Briefly, 100 μg of PRP-Exos were incubated with a staining solution mixture (0.4 μL of probe dissolved in 100 μL dilution) for 5 min at 37 °C, followed by 15 min at 4 °C in the dark. Unbound dye was subsequently removed via centrifugation (100,000×*g*, 70 min). The TSPCs were incubated with fluorescently labeled PRP-Exos (50 μg/mL) for 24 h. Following fixation with 4% paraformaldehyde fixation (BN20094, Biorigin, China), cells were stained with DAPI (C1005, Beyotime, Beijing, China). The internalization of PRP-Exos by TSPCs was assessed using laser confocal microscopy (FV1000, Olympus, Tokyo, Japan).

### Proliferation analysis of TSPCs

CCK-8 assay: TSPCs (4 × 10^3^ cells) were inoculated into each well of a 96-well plate. After 24 h, 100 μL of PRP-Exos medium (0, 10, 20, 50, 80, and 110 μg/mL) was added to each well. Following a subsequent 24 h, 48 h, and 72 h incubation period, the medium was replaced with a solution containing 10% CCK-8 reagent (BN15201, Biorigin, China) reagent and further incubated for 1 h. The optical density was then measured at a wavelength of 450 nm using a multifunctional microplate reader (Tecan, Switzerland).

EdU staining assay: To assess the effect of PRP-Exos on TSPC proliferation, we uniformly seeded 2 × 10^4^ cells/well in 24-well plates equipped with cell-climbing plates. Subsequently, the cells were allowed to adhere overnight. Following a 48 h exposure to varying concentrations of PRP-Exos (0, 20, and 50 μg/mL), EdU (C0071S, BeyoClick™ EdU Cell Proliferation Kit with Alexa Fluor 488, Beyotime) was added to stain the cells for 6 h, following the manufacturer’s provided protocol. Subsequently, the cells were fixed with 4% paraformaldehyde, and the cell nuclei were stained with DAPI (Beyotime). Fluorescence microscopy (ZOE Fluorescent Cell Imager, Bio-rad, CA, USA) was employed to capture images of EdU-positive cells. In each group, five fields of view were randomly chosen for counting. The ratio of proliferating cells was determined by dividing the count of EdU-positive cells by the total cell count in each respective field of view.

### Analysis of TSPCs migration

Transwell assay: The transwell co-culture system was employed to evaluate the effect of PRP-Exos on TSPCs migration. A total of 5 × 10^4^ cells/well were uniformly seeded in the upper chamber of transwell plates (8-μm pore size, six-well format, Corning, USA), and 500 μL of serum-free medium was added. In the lower chamber, 600 μL of PRP-Exos medium (supplemented with 10% FBS, Thermo Scientific) at varying concentrations (0, 20, and 50 μg/mL) was introduced. Following 24 h of incubation, non-migrated cells in the upper layer of the upper chamber were gently wiped off, and the migrated cells on the permeable membrane were fixed with 4% paraformaldehyde (Biorigin) and subsequently stained with crystal violet. Using an inverted microscope (Nikon, Japan), we examined five randomly selected fields of view and counted the number of migrated cells.

Scratch wound-healing assay: TSPCs (5 × 10^4^ cells) were uniformly seeded in six-well plates. When the cells' confluency achieved 80–90% confluency, a straight and consistent scratch across the cell monolayer was created using a P200 pipette tip. Subsequently, cell debris was removed by rinsing with PBS, and 2 mL of serum-free medium containing varying concentrations of PRP-Exos was introduced into each well. Cell migration was documented at 0 and 24 h for all cell groups using an inverted microscope (Nikon).

### QRT-PCR analysis

TSPCs were uniformly seeded at a density of 5 × 10^4^ cells in six-well plates and cultured overnight to promote adherence. Subsequently, after 3, 5, and 7 days of cell stimulation with PRP-Exos (0 and 50 μg/mL), total RNA was extracted from each experimental group using Trizol reagent (15596026CN, Invitrogen, USA). Total RNA was then reverse-transcribed into cDNA using the iScript cDNA Synthesis Kit (#1708891, Bio-rad), following the manufacturer’s protocol. The mRNA expression levels of the genes *COL1A1*, *SCX*, *COL2A1*, and *SOX9* were quantified using specific primers. *GAPDH* mRNA served as an internal reference to normalize mRNA quantities. Primer sequences can be found in Supplementary Table S1.

### Preparation and assessment of FG-PRP-Exos

Preparation of FG-PRP-Exos: PRP-Exo-loaded FG (approval number S20030070, Fibrin Sealant Kit, FIBINGLURAAS, Shanghai, China) was prepared in accordance with the manufacturer’s provided protocol. Briefly, a 25 μL suspension of PRP-Exos (20 μg/μL) was thoroughly mixed with 25 μL of fibrinogen (100 mg/mL) to create an exosome-fibrinogen mixture. Subsequently, this mixture was combined in a 1:1 ratio with 50 μL of thrombin (500 IU/mL) using a double syringe. After a brief incubation period of 5–8 s, the FG-PRP-Exos gel was formed, with a final concentration of 500 μg of PRP-Exos per gel.

Distribution of PRP-Exos encapsulated in FG: An aliquot of 100 μL of FG-PRP-Exos labeled with PKH67 dye was prepared and applied to a glass slide. The distribution of exosomes was observed via laser scanning confocal microscopy (FV3000, Olympus).

In vitro assessment of sustained release and exosome retention in FG-PRP-Exos: A previously established method was followed with modifications as described in refs. ^45,46^. DiR dye (BN14006, Biorigin)-labeled FG-PRP-Exos (100 μL) were incubated in a 48-well plate containing 500 μL of PBS at specific time intervals (37 °C). At 0, 3, 9, 18, 36, and 72 h, the supernatant was collected, and the fluorescence signal intensity of the exosomes in the supernatants was assessed using the IVIS Lumina Imaging System (PerkinElmer). By establishing a linear correlation between exosome content and fluorescence signal intensity, we computed the sustained-release quantity and retention rate of PRP-Exos.

A standard curve was established for exosome-constant fluorescence signal intensity: 100 μL DiR dye-labeled PRP-Exos (0, 1.56, 3.12, 6.25, 12.5, 25, and 50 μg) were placed in separate wells of a 96-well plate. The fluorescence signal intensity of exosomes in each group was measured using the IVIS Lumina Imaging System (PerkinElmer), and a correlation standard curve was plotted.

### Animals

Adult male, New Zealand White rabbits weighing 2.5 to 3.0 kg were procured from Beijing Jinmuyang Experimental Animal Breeding Co., Ltd. (SCXK(Jing) 2020-0003). The rabbits were individually housed under standard room-temperature conditions with a 12-h light/dark cycle. A total of 54 rabbits were included in this study. Twelve rabbits (24 shoulders) were randomly allocated into two groups, with each group comprising 12 shoulders, to evaluate the efficacy of the FG-PRP-Exos sustained-release system in preserving the rotator cuff injury site. The remaining 42 rabbits (84 shoulders) were randomly divided into four groups, with each group containing 21 shoulders, for in vivo investigations of acute RCT repair using PRP-Exos. These groups were as follows: (1) Model group, which involved the induction of an acute RCT model without any treatment; (2) FG group, in which FG was employed for RCT treatment; (3) FG-PRP-Exos (PRP-Exos was encapsulated in FG) group, in which a combination of FG and PRP-Exos was utilized for RCT treatment; and (4) Sham group (the Sham Control group), in which the infraspinatus tendon was exposed, but no further surgical procedure was performed.

### Surgery protocol

All rabbits were weighed and then anesthetized using a 3% pentobarbital sodium solution (1 mL/kg). Surgeries were performed under sterile conditions by two physicians. Following bilateral shoulder skin preparation and disinfection with iodophor, a model of bilateral upper extremity acute RCT was established. A 3-cm incision was created along the dorsal side of the rabbit’s shoulder, and the deltoid muscle was bluntly separated along its longitudinal axis. Subsequently, the infraspinatus tendon was meticulously freed (Fig. 4a1), and two 3-0 Ethibond sutures were passed over the tendon segment (Fig. 4a2). The distal portion of the tendon was excised near the insertion point, and the tendon fibers at the insertion site were scraped clean. A 5-mL needle was passed through the humerus to construct a parallel double-row bone medullary canal (Fig. 4a3), and two sutures were cautiously threaded through the bone tunnel (Fig. 4a4).

Subsequently, distinct interventions were performed based on the groupings. (1) Model group: Following the cutting of the infraspinatus tendon, immediate suturing was performed, and no therapeutic drugs were administered. (2) FG group: Following the incision of the infraspinatus tendon, a mixture of 50 μL of fibrinogen (50 mg/mL) and 50 μL of thrombin (500 IU/mL) in a 1:1 ratio was injected at the BTI notch using a double syringe. After the FG was formed, the infraspinatus tendon was covered over the gel and sutured. (3) FG-PRP-Exos group: After the infraspinatus tendon was cut, a 50 μL mixture of PRP-Exos and fibrinogen, along with 50 μL of thrombin solution (please see “Preparation and assessment of FG-PRP-Exos”), was injected in situ using a double syringe to establish an FG-PRP-Exos sustained-release system (containing 500 μg of PRP-Exos per shoulder). Subsequently, the infraspinatus tendon was covered with gel and sutured.

To attain optimal gel formation, we performed in situ injection gumming for 5–8 s. Following these procedures (Figs. 4a5 and 6), the tendon was subsequently sutured using the Mason–Allen technique (Fig. 4a8), and the incisions were closed, layer by layer.

### Evaluation of the in-situ retention of exosomes in vivo

In the PRP-Exos group, 100 μL of DiR-labeled PRP-Exos (5 μg/μL) was injected at the BTI. In the FG-PRP-Exos group, 100 μL DiR-labeled exosome-gel was administered following the surgical protocol described earlier. Ex vivo samples of rotator cuff tissues were collected at various time intervals post surgery (0, 3, 7, and 14 days). The fluorescence signal intensity of these samples was quantified using the IVIS Lumina Imaging System (PerkinElmer).

### Biomechanics assessment

A biomechanics tester (ShenCe, Guangdong, China) was employed for the biomechanical assessment of rotator cuff specimens 12 weeks post surgery. These rotator cuff samples were obtained and positioned on the testing apparatus. The fixture separation distance was maintained at 24 mm, and the specimen was subjected to a 5 N force for 10 min, followed by tension at a rate of 1 mm/s until rupture. Efforts were made to minimize the torsion of the tendon at the BTI. The recorded parameters included the failure load (N), relative displacement (△*x*, mm), and tendon cross-sectional area (mm^2^), from which the ultimate failure load, stiffness, and ultimate stress were derived.

### Gross evaluation of subdeltoid adhesion grading

A modified grading system (Supplementary Table S2) was employed to assess the extent of subdeltoid adhesion at the repair site, with evaluation performed independently by two observers^47,48^.

### Micro-CT analysis

A micro-CT system (SkyScan 1176, Kontich, Belgium) was utilized to evaluate the extent of new bone formation at 8 and 12 weeks post surgery at the BTI. Rotator cuff samples were subjected to scanning using the following parameters: an X-ray voltage of 70 kV and a current of 114 mA. Subsequently, these scans were reconstructed in 3D. The region of interest (ROI) was defined as a cube of 5 mm × 5 mm^2^, encompassing the entire area of the humeral greater tubercle. The bone volume fraction (BV/TV) was subsequently calculated.

### Ultrasound evaluation of the infraspinatus muscle

At 8 and 12 weeks post surgery, we conducted conventional ultrasound assessments of the infraspinatus muscle using the Aixplorer Ultrasound System (equipped with the L10-2 linear array probe, SuperSonic Imagine, France). Images of the thickest section of the muscle along its short axis were acquired, with careful delineation of the muscle boundary to measure the largest cross-sectional area. Longitudinal section images of the muscle were captured for analyzing muscle texture and assessing fat infiltration. MATLAB software (version R2020b, MathWorks, Natick, USA) was employed to extract muscle texture features, quantifying two distinct gray-level co-occurrence matrix features: ASM and IDM. Additionally, we utilized SWE imaging to evaluate the stiffness of the muscle. Longitudinal section images of the thickest part of the infraspinatus muscle were obtained, and after the images were stabilized, a circular ROI with a 5 mm diameter was selected for measuring the shear wave velocity value. We took care to exclude bones and fascia from the ROI. Each rotator cuff sample underwent three measurements.

### Histological assessment

The rotator cuff samples were collected at 8 and 12 weeks post surgery. Subsequently, a series of steps, including fixation, decalcification, dehydration, and paraffin embedding, were performed. Coronal sections of the BTI with a maximum thickness of 6 μm were obtained. These sections were subjected to staining procedures, including Hematoxylin–eosin staining, Masson trichrome staining, and safranin-O staining were performed. The stained tissue sections were examined using a virtual panoramic microscope (Olympus) and ImageJ software (version 2.3.0, NIH, Bethesda, USA).

### Statistics and reproducibility

Sample sizes of animal experiments were performed using Power Analysis and Sample Size software (PASS, version 15.0.5, NCSS, USA), and the sample sizes were presented in the figure legends. All data were presented as means ± standard deviation, and the data were statistically analyzed using GraphPad Prism 9 (version 9.5.0, GraphPad Software, CA, USA). The normal distribution of data was assessed using the Kolmogorov–Smirnov test or Shapiro–Wilk test. When the data exhibited a normal distribution, and the variance was homogeneous, differences between two independent groups were assessed using the two-tailed unpaired Student’s *t* test, whereas the one-way analysis of variance with Tukey’s multiple-comparison test was employed for comparisons involving multiple groups. Ranked data were analyzed using the Kruskal–Wallis test. A significance threshold of *P* < 0.05 was applied to determine statistical significance. All data were obtained from three independent replicates to mitigate the influence of chance (*P < 0.05, **P < 0.01, ***P < 0.001).

### Reporting summary

Further information on research design is available in the Nature Portfolio Reporting Summary linked to this article.

### Supplementary information

Supplementary Information

Description of Supplementary Materials

Supplementary Data

Reporting Summary

## Data Availability

The source data for the graphs in this study are provided in Supplementary Data. The Western blot uncropped images are provided in Supplementary Fig. 1 in Supplementary Information. All other supporting data generated and/or analyzed during this study are available from the corresponding author upon reasonable request.
