# Supplementary material for: In-situ gelation of fibrin gel encapsulating platelet-rich plasma-derived exosomes promotes rotator cuff healing
Source: Commun Biol. 2024 Feb 20;7:205. doi: 10.1038/s42003-024-05882-7 (PMC10876555; doi:10.1038/s42003-024-05882-7)
Supplement: Supplementary file 1 — Supplementary Information [file 42003_2024_5882_MOESM1_ESM.pdf]

Supplementary Information

In-Situ Gelation of Fibrin Gel Encapsulating Platelet-Rich Plasma-Derived Exosomes Promotes Rotator Cuff Healing

Molin Li[1,2,3,*], Lin Shi[4,*], Xianghui Chen[2], Dan Yi[2], Yufei Ding[4], Jian Chen[2,5], Guanghui Xing[6], Siming Chen[2], Li Wang[1], Yongyi Zhang[1,7], Yaqiong Zhu[2,#], Yuexiang Wang[2,#]

[1] Medical School of Chinese PLA, Beijing 100853, China.

[2] Department of Ultrasound, The First Medical Center, Chinese PLA General Hospital, Beijing 100853, China.

[3] State Key Laboratory of Kidney Diseases, National Clinical Research Center for Kidney Diseases, Beijing Key Laboratory of Kidney Disease Research, Beijing 100853, China.

[4] Beijing Friendship Hospital, Capital Medical University, Beijing 100050, China.

[5] School of Medicine, Nankai University, Tianjin 300071, China.

[6] Department of Ultrasound, the Fourth Medical Center, Chinese PLA General Hospital, Beijing 100048, China.

[7] No.962 Hospital of the PLA Joint Logistic Support Force, Harbin 150080, China

[*] **These authors contributed equally: Molin Li and Lin Shi.**

[#] **These authors jointly supervised this work: Yuexiang Wang and Yaqiong Zhu.**

[#] **Correspondence: Yuexiang Wang, E-mail: wangyuexiang1999@sina.com**

[#] **Correspondence: Yaqiong Zhu, E-mail: zhuyaqiong28@163.com**

Supplementary Fig. 1

Unedited/uncropped western blots for Fig. 1c

CD 9

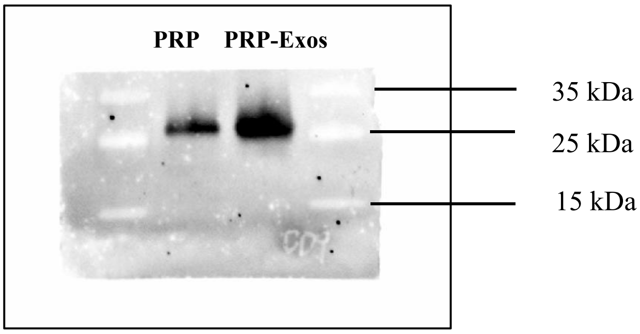

CD 63

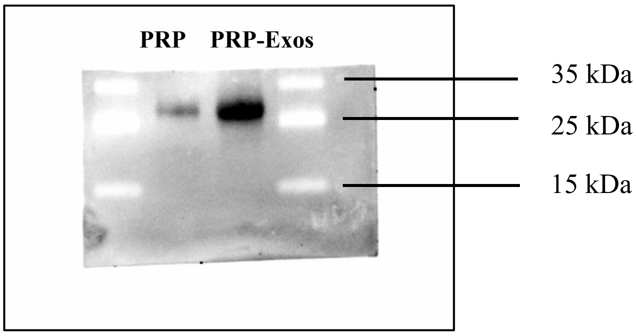

CD 81

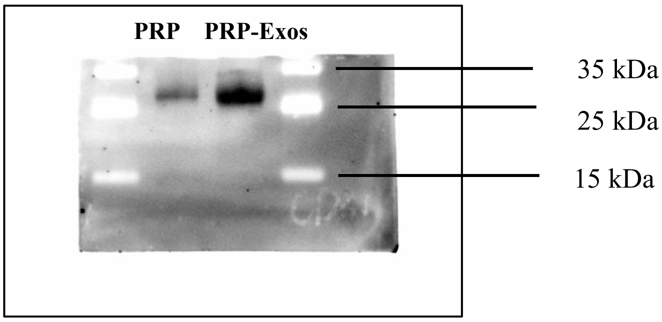

CD 41

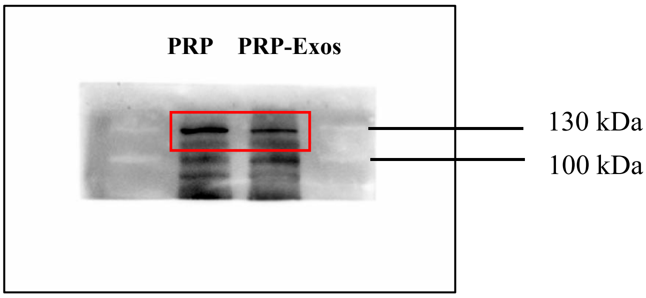

Supplementary Table S1

Primer sequences used for polymerase chain reactions.

Gene	Sequence
COL1A1	Forward: 5’-CACTGCAAGAACAGCGTAGC-3’
	Reverse: 5’-AAGTTCCGGTGTGACTCGTG-3’
SCX	Forward: 5’-CTGTGAACAGAGAGATGGACAG-3’
	Reverse: 5’-GTAGAGAGCCAGCATGGAAAG-3’
COL2A1	Forward: 5’-GAGTGGAAGAGCGGAGACTACTG-3’
	Reverse: 5’-GTCTCCATGTTGCAGAAGACTTTCA-3’
SOX9	Forward: 5’-CCAGCAAGAACAAGCCACAC-3’
	Reverse: 5’-CTTGCCCAGAGTCTTGCTGA-3’
GAPDH	Forward: 5’-GTTCCAGTATGACTCTACCCACG-3’
	Reverse: 5’-CATTTGATGTTAGCGGGATCTCG-3’

Supplementary Table S2

A modified scoring system for overall scoring of subdeltoid adhesions.

Grade	Adhesion Extent
0	No adhesion
1	Adhesion separated with gentle retraction
2	Adhesion separated with moderate retraction
3	Adhesion separated with sharp retraction
4	Adhesion with fixed fibrotic tissue
