# Supplementary material for: In-situ gelation of fibrin gel encapsulating platelet-rich plasma-derived exosomes promotes rotator cuff healing
Source: Commun Biol. 2024 Feb 20;7:205. doi: 10.1038/s42003-024-05882-7 (PMC10876555; doi:10.1038/s42003-024-05882-7)
Supplement: Supplementary file 2 — Description of Supplementary Materials [file 42003_2024_5882_MOESM2_ESM.docx]

**Description of Additional Supplementary Files**

**File name:** Supplementary Data 1

**Description:** The source data behind all the graphs in the paper.
