# Supplementary material for: In-situ gelation of fibrin gel encapsulating platelet-rich plasma-derived exosomes promotes rotator cuff healing
Source: Commun Biol. 2024 Feb 20;7:205. doi: 10.1038/s42003-024-05882-7 (PMC10876555; doi:10.1038/s42003-024-05882-7)
Supplement: Supplementary file 4 — Reporting Summary [file 42003_2024_5882_MOESM4_ESM.pdf]

nature portfolio

Corresponding author(s): Yuexiang Wang and Yaqiong Zhu
Last updated by author(s): Jan 14, 2024

Reporting Summary

Nature Portfolio wishes to improve the reproducibility of the work that we publish. This form provides structure for consistency and transparency in reporting. For further information on Nature Portfolio policies, see our Editorial Policies and the Editorial Policy Checklist.

Statistics

For all statistical analyses, confirm that the following items are present in the figure legend, table legend, main text, or Methods section.

n/a	Confirmed	
☐	☒	The exact sample size (*n*) for each experimental group/condition, given as a discrete number and unit of measurement
☐	☒	A statement on whether measurements were taken from distinct samples or whether the same sample was measured repeatedly
☐	☒	The statistical test(s) used AND whether they are one- or two-sided<br>*Only common tests should be described solely by name; describe more complex techniques in the Methods section.*
☒	☐	A description of all covariates tested
☒	☐	A description of any assumptions or corrections, such as tests of normality and adjustment for multiple comparisons
☐	☒	A full description of the statistical parameters including central tendency (e.g. means) or other basic estimates (e.g. regression coefficient) AND variation (e.g. standard deviation) or associated estimates of uncertainty (e.g. confidence intervals)
☒	☐	For null hypothesis testing, the test statistic (e.g. *F*, *t*, *r*) with confidence intervals, effect sizes, degrees of freedom and *P* value noted<br>*Give P values as exact values whenever suitable.*
☒	☐	For Bayesian analysis, information on the choice of priors and Markov chain Monte Carlo settings
☒	☐	For hierarchical and complex designs, identification of the appropriate level for tests and full reporting of outcomes
☒	☐	Estimates of effect sizes (e.g. Cohen's *d*, Pearson's *r*), indicating how they were calculated

Our web collection on statistics for biologists contains articles on many of the points above.

Software and code

Policy information about availability of computer code

Data collection	No software was used.
Data analysis	the GLCM features were calculated using functions provided by Matlab R2020b. Other data were analyzed using GraphPad Prism9.

For manuscripts utilizing custom algorithms or software that are central to the research but not yet described in published literature, software must be made available to editors and reviewers. We strongly encourage code deposition in a community repository (e.g. GitHub). See the Nature Portfolio guidelines for submitting code & software for further information.

Data

Policy information about availability of data

All manuscripts must include a data availability statement. This statement should provide the following information, where applicable:

- Accession codes, unique identifiers, or web links for publicly available datasets
- A description of any restrictions on data availability
- For clinical datasets or third party data, please ensure that the statement adheres to our policy

All data generated and/or analyzed during this study are available from the corresponding author upon reasonable request.

Research involving human participants, their data, or biological material

Policy information about studies with human participants or human data. See also policy information about sex, gender (identity/presentation), and sexual orientation and race, ethnicity and racism.

Reporting on sex and gender	This study involves only the collection of whole blood from healthy volunteers for the extraction of PRP, and all volunteers have signed informed consent forms. It should be emphasized that this is a basic research study, not primarily focusing on human subjects. Therefore, we did not consider the potential impact of sex on the research results. The sex of the volunteers was selected randomly, and we did not conduct a sex analysis.
Reporting on race, ethnicity, or other socially relevant groupings	This study does not involve this issue.
Population characteristics	This study does not involve this issue.
Recruitment	We recruited healthy volunteers for this study. The selection criteria were based on age, general health status, and the absence of any known chronic diseases. All participants were informed about the purpose of the study and provided their informed consent.
Ethics oversight	Ethics Committee Of Chinese PLA General Hosptial

Note that full information on the approval of the study protocol must also be provided in the manuscript.

Field-specific reporting

Please select the one below that is the best fit for your research. If you are not sure, read the appropriate sections before making your selection.

☒ Life sciences ☐ Behavioural & social sciences ☐ Ecological, evolutionary & environmental sciences

For a reference copy of the document with all sections, see nature.com/documents/nr-reporting-summary-flat.pdf

Life sciences study design

All studies must disclose on these points even when the disclosure is negative.

Sample size	A total of 108 rotator cuffs were included in this study. Of these, 24 rotator cuffs were randomly divided into two groups for the evaluation of the in vivo retention capacity of FG-PRP-Exos; the remaining 84 rotator cuffs were randomly divided into four groups for the related evaluation of the therapeutic effect of FG-PRP-Exos. The determination of the sample size was based on the results of the preliminary experiment and referred to the sample size in previous literature, ensuring sufficient samples were included in the study.
Data exclusions	No data were excluded from the analyses.
Replication	All experiments were performed in triplicate, and yielded consistent trends.
Randomization	All samples were randomly assigned to groups.
Blinding	During image acquisition and result analysis, the investigators were blind to the group assignments.

Reporting for specific materials, systems and methods

We require information from authors about some types of materials, experimental systems and methods used in many studies. Here, indicate whether each material, system or method listed is relevant to your study. If you are not sure if a list item applies to your research, read the appropriate section before selecting a response.

Materials & experimental systems

n/a	Involved in the study	
☐	☒	Antibodies
☐	☒	Eukaryotic cell lines
☒	☐	Palaeontology and archaeology
☐	☒	Animals and other organisms
☒	☐	Clinical data
☒	☐	Dual use research of concern
☒	☐	Plants

Methods

n/a	Involved in the study	
☒	☐	ChIP-seq
☒	☐	Flow cytometry
☒	☐	MRI-based neuroimaging

Antibodies

Antibodies used	CD9 Monoclonal antibody 60232-1-Ig, Proteintech<br>CD63 Monoclonal antibody 67605-1-Ig, Proteintech<br>CD81 Monoclonal antibody 66866-1-Ig, Proteintech<br>CD41/Integrin Alpha 2B Monoclonal antibody 60350-1-Ig, Proteintech<br>CD44 Polyclonal antibody 15675-1-AP, Proteintech
Validation	The species for CD9, CD63, CD81, and CD41 is mouse, and all antibodies were verified to react with humans according to official website information, and were used for Western Blot experiments.<br>The species for CD44 is rabbit, and It was verified to react with rats, and was applied in immunofluorescence (IF) experiments.

Eukaryotic cell lines

Policy information about cell lines and Sex and Gender in Research

Cell line source(s)	The tendon stem/progenitor cells we extracted were derived from male suckling rats.
Authentication	Immunofluorescence staining was used to identify the characteristic marker protein CD44 in tendon stem/progenitor cells.
Mycoplasma contamination	The cell lines were not tested for mycoplasma contamination.
Commonly misidentified lines<br>(See ICLAC register)	No commonly misidentified cell lines were used.

Animals and other research organisms

Policy information about studies involving animals; ARRIVE guidelines recommended for reporting animal research, and Sex and Gender in Research

Laboratory animals	New Zealand rabbits, weighing between 2.5 to 3 kg, were male.
Wild animals	This study did not involve wild animals.
Reporting on sex	This study utilized male New Zealand rabbits, and the results of the research were independent of the sex of the experimental animals.
Field-collected samples	This study did not involve samples collected from the field.
Ethics oversight	All procedures described in this study were approved by the Ethics Committee of the Chinese PLA General Hospital.

Note that full information on the approval of the study protocol must also be provided in the manuscript.

Plants

Seed stocks	N/A
Novel plant genotypes	N/A
Authentication	N/A
